# Gasdermin E in glioblastoma –pyroptosis resistance and tumor-promoting functions

**DOI:** 10.1038/s41420-025-02572-z

**Published:** 2025-06-21

**Authors:** Ege Solel, Egil Brudvik, Lars Andreas Rømo Ystaas, Yahaya A. Yabo, Emma Rigg, Romi Roy Choudhury, Halala Sdik Saed, Dieter Henrik Heiland, Rolf Bjerkvig, Jubayer Hossain, Hrvoje Miletic

**Affiliations:** 1 Department of Biomedicine, University of Bergen, Bergen, Norway; 2 Institute for Clinical Medicine, University of Oslo, Oslo, Norway; 3 Department of Neurosurgery, Medical Center, Faculty of Medicine, Erlangen University, Erlangen, Germany; 4 Comprehensive Cancer Center Freiburg (CCCF), Medical Center, University of Freiburg, Freiburg, Germany; 5 German Cancer Consortium (DKTK) partner site Freiburg, Freiburg, Germany; 6 Department of Neurological Surgery, Lou and Jean Malnati Brain Tumor Institute, Robert H. Lurie Comprehensive Cancer Center, Feinberg School of Medicine, Northwestern University, Chicago, IL USA; 7 Faculty of Nursing and Health Sciences, Nord University, Namsos, Norway; 8 Department of Pathology, Haukeland University Hospital, Bergen, Norway

**Keywords:** CNS cancer, Cancer microenvironment, Cancer therapeutic resistance

## Abstract

Treatment of glioblastoma (GB), the most common and most aggressive malignant brain tumor, has made little progress over the past two decades. Despite extensive research on apoptosis and autophagy, necrotic cell death mechanisms like pyroptosis, which have the potential to stimulate anti-tumor immune responses, remain largely underexplored in GB. Here, we investigated whether Gasdermin E (GSDME)-mediated pyroptosis can be induced in GB by employing the drug raptinal, an inducer of cytochrome c release. Using human patient-derived and mouse GB cell lines, we showed that raptinal promotes GSMDE cleavage. However, although a strong pyroptotic response was observed in mouse cell lines, it was weak in human cell lines. This resistance was partially reversed by the calcium chelator BAPTA-AM, indicating that membrane repair mechanisms may counteract the pyroptotic response. *Gsdme* knockout (KO) in mouse GB cells unexpectedly prolonged the survival of immunocompetent mice, demonstrating a tumor-promoting role of GSDME independent of its pyroptotic function. Analysis of the immune microenvironment revealed that *Gsdme* KO promoted infiltration of T cells, which was confirmed by spatial transcriptomic analysis of GB patient samples. In addition, *Gsdme/GSMDE* KO reduced the invasive capacity of mouse/human GB cells. In conclusion, active membrane repair mechanisms may impair the pyroptotic efficacy in GB. GSDME has a tumor-promoting role in GB by suppressing T cell infiltration and increasing tumor cell invasion.

## Introduction

Glioblastoma (GB) is the most aggressive and most frequent primary brain tumor, diffusely infiltrating into the brain and promoting excessive angiogenesis [1]. Single cell sequencing data, including spatial transcriptomics, have revealed extensive heterogeneity within single tumors, emphasizing a high degree of intra- and intertumoral heterogeneity [2]. Despite the detailed characterization of GB landscapes at genomic, epigenetic, transcriptomic, and metabolic levels, little progress has been made regarding the development of more efficient treatment modalities. One important molecular factor for treatment outcome is the type of cell death that tumor cells undergo and the impact on the tumor immune microenvironment. While apoptosis and autophagy are very well explored in GB [3], other, in particular, necrotic types of cell death are less characterized. These have been explored to a larger extent in other solid cancers, such as melanoma, revealing therapeutic opportunities by activating an anti-tumor immune response [4, 5].

Pyroptosis is a necrotic type of cell death mediated by the proteins of the Gasdermin family [6]. Gasdermins are cleaved by caspases to induce pore formation in the cellular membrane, leading to necrotic cell death [7], with potential release of antigens into the environment. The pyroptotic function of Gasdermin E (GSDME) was discovered in 2017 by Wang et al., demonstrating that GSDME was cleaved by caspase 3 when cancer cells were treated with chemotherapy drugs. The authors also observed that *GSDME* was silenced in cancer cells but expressed in normal cells [8]. This was confirmed by Zhang et al., showing that *GSDME* expression is suppressed in cancers due to frequent mutations [4]. When *GSDME* was knocked out in cancer cells, tumor growth was enhanced. This tumor suppressive function of GSDME was attributed to increased phagocytosis of tumor cells by macrophages and increased infiltration of natural killer cells and CD8+ tumor cells, contributing to anti-tumor immunity [4]. Thus, delivering GSDME or potentially other Gasdermins with similar functions is a promising strategy to enhance anti-tumor immunity.

In this study, we investigated the pyroptotic function of GSDME in GB and discovered that human GB shows resistance to pyroptosis despite successful GSDME activation. In addition, we identified tumor-promoting functions of GSDME in GB that are independent of pyroptosis.

## Results

### GSDME is expressed in GB at high levels

**Fig. 1:**
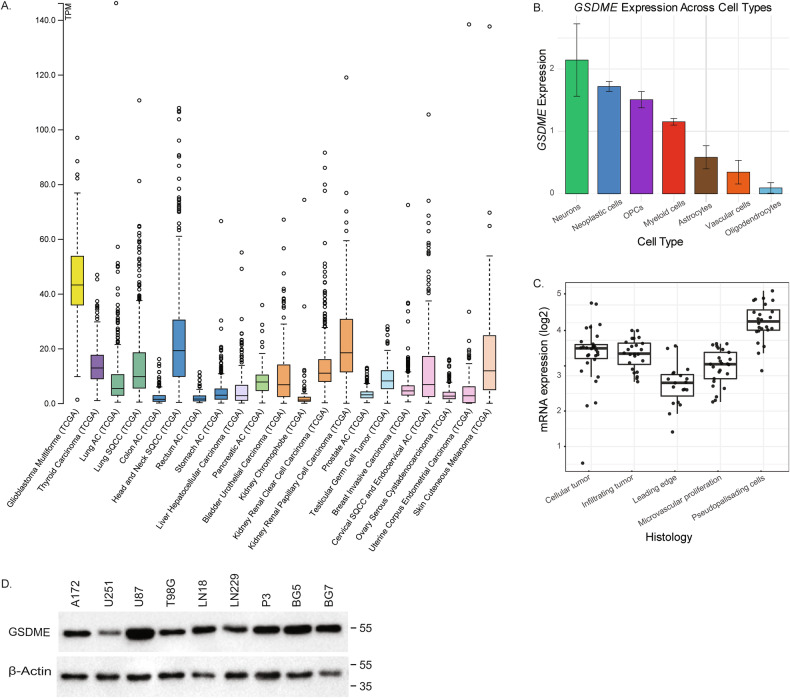
GSDME is expressed in GB at high levels. **A** TCGA data extracted from proteinatlas.org [10] shows higher expression levels of *GSDME* in GB compared to other cancer types. RNA expression overview demonstrates RNA-seq data from TCGA, with expression levels presented as Transcripts Per Million (TPM) on the *y*-axis. **B** *GSDME* is highly expressed in GB cells, with levels comparable to those found in neurons and oligodendrocyte precursor cells (OPCs). Data derived Darmanis et al. [11]. **C** Analysis of histologically defined regions in GB from the Ivy GB Atlas [12] reveals the highest expression of *GSDME* in regions around necrosis with pseudopalisading cells. **D** Western blots of human GB cell lines and GSC lines show high GSDME expression in all samples. β-actin was used as loading control.

*GSDME* is regarded as a tumor suppressor gene, and it has been shown that many cancers harbor inactivating mutations [4]. Thus, we first analyzed *GSMDE* expression and mutation rate in GB in public datasets. Analysis of TCGA data using cBioportal [9] revealed that *GSDME* was amplified in 4% of GB patients, and only 1 out of 368 patients showed a mutation, which was a splice mutation of unknown significance. Analysis of TCGA data across cancers in proteinatlas.org [10] showed that *GSDME* was expressed at higher levels in GB compared to other cancers (Fig. 1A). Analysis of single-cell RNA sequencing data from Darmanis et al. [11] also showed a high-level expression of *GSDME* in tumor cells, which was comparable to the levels found in neurons and oligodendrocyte precursor cells (Fig. 1B). However, GSDME expression in the tumor core was higher compared to the tumor periphery and distant areas (Supplementary Fig. 1A). When analyzing GB cell subpopulations as categorized by Neftel et al. in the spatial transcriptomics GBmap dataset [2], no differences in *GSDME* expression were found between AS-like, MES-like, OPC-like, and NPC-like subsets (Supplementary Fig. 1B). Analysis of characteristic histologically defined GB regions from the Ivy GB Atlas [12] showed the highest expression of *GSDME* in pseudopalisading cells and the lowest expression at the leading edge (Fig. 1C). We confirmed high expression levels of GSDME in human GB cell lines as well as patient-derived GB stem cell (GSC) lines by western blot (Fig. 1D, Supplementary original western blots). When GB/GSC cell lines were cultured under hypoxic or normoxic conditions, GSDME expression levels did not change substantially (Supplementary Fig. 1C, Supplementary material). This was confirmed by analyzing the spatial transcriptomics GBmap dataset, showing that *GSDME* expression was not influenced by hypoxic regions (Supplementary Fig. 1D). In conclusion, GSDME is expressed at high levels in GB cells.

### Raptinal induces cytochrome c release and GSDME cleavage in GB cell lines

**Fig. 2:**
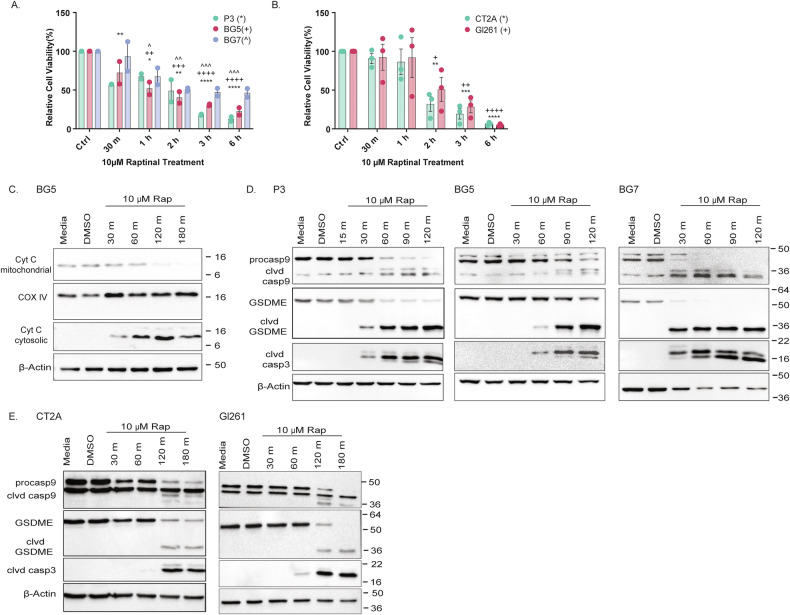
Raptinal induces cytochrome c release and GSDME cleavage in GB cell lines. **A** WST-1 assay of human GSC lines treated with 10 μM raptinal. Time points after the start of treatment are indicated. Data represented as mean ± SEM (N = 2). ** (and corresponding signs) *p* < 0.01; *** (and corresponding signs) *p* < 0.001; *** (and corresponding signs) *p* < 0.0001. **B** WST-1 assay of mouse GB lines treated with 10 μM raptinal. Time points after the start of treatment are indicated. Data represented as mean ± SEM (N = 3). * (and corresponding signs) p < 0.05; ** (and corresponding signs) *p* < 0.01; *** (and corresponding signs) *p* < 0.001; *** (and corresponding signs) *p* < 0.0001. **C** Western blot of cytosolic and mitochondrial cytochrome C in BG5 cells treated with 10 μ μM raptinal. COXIV was used as a mitochondrial loading control, and β-actin as a cytosolic loading control. **D** Western blot for cleaved (clvd) Caspase 3, Caspase 9, clvd Caspase 9, GSDME, and clvd GSDME in GSC lines treated with 10 μM raptinal. Time points after the start of treatment are indicated. β-actin was used as loading control. **E** Western blot for cleaved (clvd) Capsase 3, Caspase 9, clvd Caspase 9, GSDME, and clvd GSDME in mouse GB lines treated with 10 μM raptinal. Time points after the start of treatment are indicated. β-actin was used as loading control.

To investigate if GSDME can execute a pyroptotic program in GB cells, we used raptinal, a fast inducer of apoptosis [13], which recently has been shown to execute GSDME-mediated pyroptosis downstream of caspase 3 in melanoma cells [14]. We first analyzed the effect of raptinal on GB viability using WST-1 assay. The human GSC lines P3 and BG5 were sensitive to 10 μM raptinal treatment with complete loss of viability (Fig. 2A). The GSC line BG7 showed less sensitivity to raptinal treatment compared to P3 and BG5, with ca. 50% loss of viability. Mouse GB cell lines CT2A and GL261 were both highly sensitive to raptinal treatment with complete loss of viability (Fig. 2B). Sensitivity to raptinal was confirmed in B16F10 mouse melanoma cells, as previously reported [4], as well as in A549 and L929 cells (Suppl. Fig. 2A). Raptinal mediates the release of cytochrome c from mitochondria into the cytosol [15]. This was confirmed in BG5, P3, and GL261 cells upon raptinal treatment by western blot (Fig. 2C and Supplementary Fig. 2B, Supplementary original western blots). We then analyzed whether cytochrome c release initiated the intrinsic apoptotic cascade with consecutive caspase 9 and caspase 3 cleavage. We confirmed cleavage of both caspases in human and mouse GB lines (Fig. 2D, E; Supplementary material) at different time points indicated. Activated Caspase 3 cleaves GSDME to induce pyroptosis [8]. We observed GSDME cleavage in all human and mouse lines. In human GSC lines, cleavage was observed already at 30 min (Fig. 2D), while cleavage was detected earliest at 120 min in mouse GB lines (Fig. 2D, E; Supplementary material). Caspase 3 and GSDME cleavage were also confirmed in A549 and B16F10 cells at 60 min, which were used as controls (Supplementary Fig. 2C, Supplementary material). Thus, both human GSC and mouse GB lines can activate pyroptotic machinery at the molecular level upon raptinal treatment.

### Human GB cells show resistance to GSDME-mediated pyroptosis

**Fig. 3:**
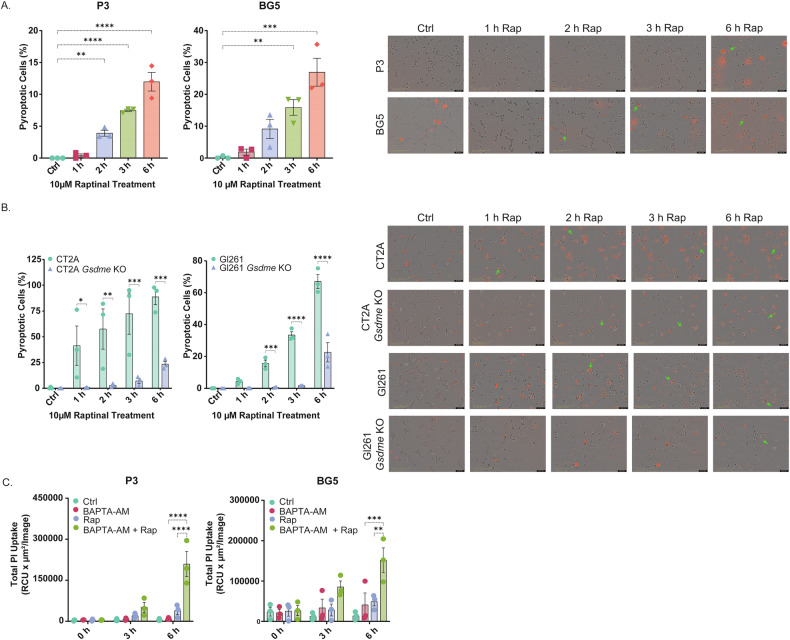
Human GB cells show resistance to GSDME-mediated pyroptosis. **A** Pyroptosis induction in human GSCs was quantified at the indicated time points post-raptinal treatment by microscopy, combining morphology and PI uptake. Data represented as mean ± SEM (*N* = 3). **p < 0.01; ***p < 0.001; ****p < 0.0001. The scale bar indicates 200 µm. **B** Pyroptosis induction in mouse GB lines was quantified at the indicated time points post-raptinal treatment by microscopy, combining morphology and PI uptake. Data represented as mean ± SEM (*N* = 3). *p < 0.05; **p < 0.01; ***p < 0.001; ****p < 0.0001. The scale bar indicates 200 µm. **C** Pyroptosis was measured by PI uptake in human GSC lines post-raptinal treatment at the indicated time points. BAPTA-AM was used as calcium chelator to block calcium-dependent plasma membrane repair mechanisms [16]. Data represented as mean ± SEM (*N* = 3). **p < 0.01; ***p < 0.001; ****p < 0.0001.

To analyze if the cleavage of GSDME results in pyroptosis in GB, we analyzed cultures of human GSC and mouse GB lines for morphological signs of pyroptosis in combination with Propidium-iodide (PI) uptake. Human GSC lines showed surprisingly low susceptibility to pyroptosis, particularly at early time points (1–3 h; Fig. 3A and Supplementary Fig. 3A), where GSDME cleavage had been observed by western blot (Fig. 2D). Pyroptotic efficacy was less than 10% in P3, less than 20% in BG5, and less than 30% in BG7 (Fig. 3A and Supplementary Fig. 3A). The fraction of pyroptotic cells increased up to 6 h but remained at relatively low levels (around 30% or below). In contrast, the mouse GB line CT2A showed prominent signs of pyroptosis upon raptinal treatment at early time points, increasing up to 6 h (Fig. 3B and Supplementary Fig. 3B), which correlated well with the occurrence of GSDME cleavage (Fig. 2E) and substantial loss in viability (Fig. 2B). Pyroptosis was significantly inhibited by CRISPR knockout (KO) of the mouse *Gsdme* gene (Fig. 3B and Supplementary Fig. 3C, Supplementary original western blots). The mouse GB line GL261 also showed more susceptibility to pyroptosis compared to the human lines, but the pyroptotic response was delayed until 3 to 6 h (Fig. 3B and Supplementary Fig. 3B). Similar to CT2A, pyroptosis was inhibited in *Gsdme* KO cells (Fig. 3B and Supplementary Fig. 3C, Supplementary material). In addition, pyroptosis in both mouse GB cell lines was quantified by measuring PI uptake only, yielding results consistent with those obtained from the combined morphology and PI analysis. (Supplementary Fig. 3D). Viability measurements showed that *Gsdme* KO significantly inhibited cell death upon raptinal treatment in CT2A compared to control (ctrl) cells (Supplementary Fig. 3E). A similar trend was observed for GL261, however, without significance. Resistance to pyroptosis in human GB was confirmed by measuring PI uptake, which was very low in all GSC lines (Fig. 3C and Supplementary Fig. 3F). We hypothesized that active plasma membrane mechanisms in human GB may interfere with pore formation during pyroptosis. As plasma membrane repair is dependent on calcium, we used the calcium chelator BAPTA-AM during raptinal treatment. PI uptake/pyroptosis was significantly increased in P3 and BG5 when using BAPTA-AM compared to control, indicating active plasma membrane repair mechanisms (Fig. 3C). This increase was not observed in BG7 (Supplementary Fig. 3F); however, this GSC line was generally less susceptible to raptinal treatment compared to the other human GSC lines (Fig. 2A).

### Knockout of *Gsdme* prolongs survival in mouse GB and promotes infiltration of T cells in the GB microenvironment

**Fig. 4:**
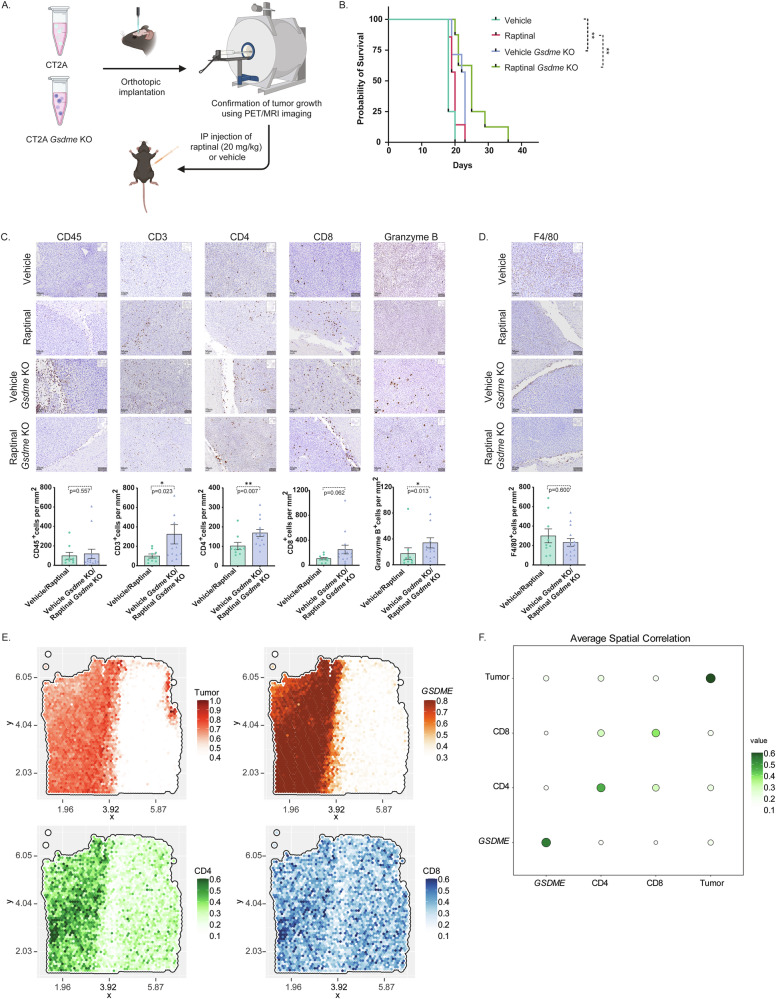
Knockout of *Gsdme* prolongs survival in mouse GB and promotes infiltration of T cells in the GB microenvironment. **A** Schematic overview of the in vivo experiment (made with Biorender). **B** Kaplan Meier survival curve (*n* = 4–8 mice/group). **P < 0.01. **C** Immunohistochemistry of FFPE tumor sections for the leukocyte antigen CD45 and T cell antigens CD3, CD4, CD8, and Granzyme B. Quantifications and analyses of pooled ctrl and *Gsdme* KO groups are shown below the images. Data represented as mean ± SEM (*n* = 3–8 mice/group). *p < 0.05; **p < 0.01. The scale bar indicates 50 µm. **D** Immunohistochemistry of FFPE tumor sections for macrophage antigen F4/80. Quantifications and analyses of pooled ctrl and *Gsdme* KO groups are shown below the images. Data represented as mean ± SEM (*n* = 3–8 mice/group). The scale bar indicates 50 µm. **E** Dot plot showing average spatial correlation between *GSDME*, GB tumor cells, CD4, and CD8 T cells. CD3D and CD3E were used as CD4 T cell markers, CD8A was used as CD8 marker, while *GFAP, SOX2, MKI67*, and *EGFR* were used as GB tumor cell markers. **F** Surface plots showing spatial expression of GSDME, GB tumor cells, CD4, and annotated CD8 T cells in one representative patient (269UKF).

Raptinal has previously been applied in vivo in melanoma and breast cancer models without signs of toxicity [13, 14]. Both studies demonstrated treatment effects either by reduction in tumor volume or prolonged survival. Raptinal is a small molecule with a molecular weight of 386.44 Da and thus should be able to cross the blood-brain barrier. To investigate if raptinal-induced pyroptosis is an efficient treatment for GB, we implanted mouse GB CT2A and CT2A *Gsdme* KO orthotopically into C57/BL6 mice. Upon tumor detection on MRI (data not shown), animals were treated with 20 mg/kg raptinal once daily for 4 days (Fig. 4A). Surprisingly, raptinal did not promote a significant treatment effect. Although mice bearing *Gsdme* knockout tumors treated with raptinal appeared to survive longer than those with vehicle-treated *Gsdme* knockout tumors, this survival advantage did not reach statistical significance. (Fig. 4B). In general, there was no significant survival difference when comparing CT2A untreated and treated as well as CT2A *Gsdme* KO untreated and treated groups (Fig. 4B). However, there was a significant increase in survival when comparing CT2A *Gsdme* KO to the CT2A group, both in untreated and treated situations. This indicates that GSDME is rather a tumor promoter than a suppressor in GB, independent of its pyroptotic function. To analyze if GSDME impacts the immune microenvironment, we immunostained histological sections with antibodies against CD45, CD3, CD4, CD8, and Granzyme B to analyze T cell infiltration (Fig. 4C). Further, we examined macrophage infiltration by F4/80 staining (Fig. 4D). Interestingly, *Gsdme* KO significantly impacted the immune microenvironment independent of raptinal treatment in a pooled analysis of control groups versus *Gsdme* KO groups (Fig. 4C). *Gsdme* KO tumors showed infiltration of significantly more CD3+, CD4+, and Granzyme B + T cells compared to ctrl tumors. A similar trend was observed for CD8 + T cells with borderline significance (*p* = 0.062). There was no significant difference in macrophage infiltration in ctrl compared to KO tumors. In multivariate sensitivity analyses, raptinal had no effect on the number of positive cells, and there were no significant interaction effects with *Gsdme* KO (Supplementary Fig. 4A). As expected, due to a higher testing burden and fewer degrees of freedom available for the test in the model, the *Gsdme* KO main effect was less statistically significant. Still, the same general pattern from the primary analysis was replicated, indicating significant differences between *Gsdme* KO and ctrl for CD3+ and CD8+ T cells (Supplementary Fig. 4A).

Next, we analyzed the distribution of T cells relative to *GSDME* expression in human GB cells in the spatial transcriptomics GBmap patient dataset of 10 patients (Suppl. Table 3) [2]. We found no spatial correlation between *GSDME* expression and both CD4+ and CD8+ T cell infiltration (Fig. 4E, F and Supplementary Fig. 4B), unlike the strong spatial correlation observed between *GSDME* expression and GB cells. TCGA dataset analysis showed anti-correlation of *GSDME* expression and CD3D, CD8A, CD8B, and Granzyme B expression (Supplementary Fig. 4C), thus confirming animal and spatial patient data. In summary, these results indicate that GSMDE remodels the tumor microenvironment by suppressing T cell infiltration.

### GSDME promotes invasion of GB cells

**Fig. 5:**
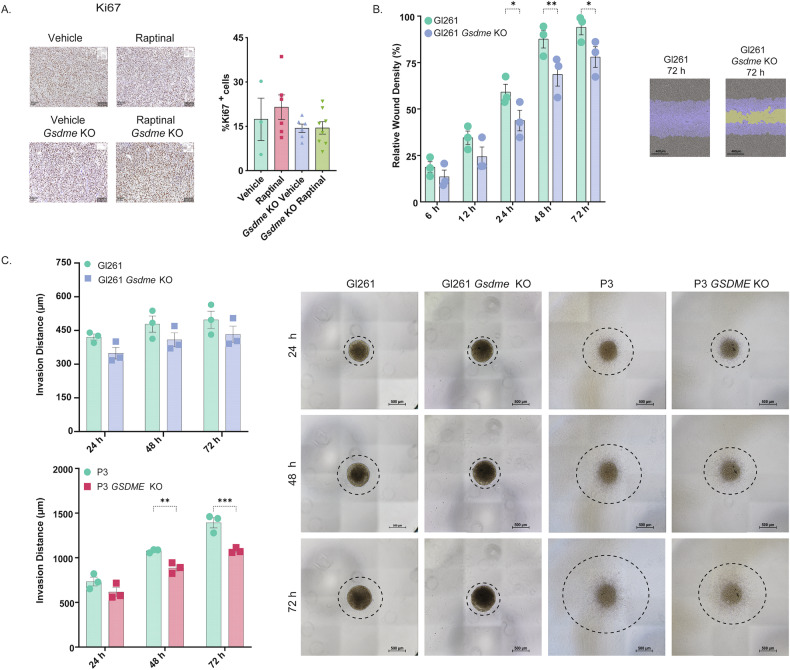
GSDME promotes invasion of GB cells. **A** Immunohistochemistry of FFPE tumor sections (in vivo experiment from Fig. 4 A) for proliferation antigen Ki67. Quantification is shown next to the images. Data represented as mean ± SEM (*n* = 3–8 mice/group). **B** Wound healing assay with GL261 and GL261 *Gsdme* KO mouse GB cells. Wound closure was quantified at indicated time points. Data represented as mean ± SEM (*N* = 3). *p < 0.05; **p < 0.01. The scale bar indicates 400 µm. **C** Collagen invasion assay of GL261 and P3 and corresponding *Gsdme*/*GSDME* KO cells. The invasion was quantified at the indicated time points. Data represented as mean ± SEM (*N* = 3). *p < 0.05. The scale bar indicates 500 µm.

Next, we aimed to analyze if GSMDE has direct tumor-promoting functions. We first analyzed Ki67 immunostainings from the in vivo experiment (Fig. 4A) and found no significant differences between the different groups (Fig. 5A). Cell cycle analysis of mouse and human GB ctrl and *Gsdme*/*GSDME* KO cells revealed no significant differences (Supplementary Fig. 5A). In contrast, wound healing assays showed significantly reduced migration of GL261 *Gsdme* KO cells compared to GL261 ctrl cells (Fig. 5B and Supplementary Fig. 5B). Collagen invasion assay of GL261 showed the same tendency (Fig. 5C). Also, human P3 *GSDME* KO cells demonstrated significantly less invasion into collagen compared to control cells (Fig. 5C). In conclusion, GSDME promotes invasion/migration of GB cells.

## Discussion

Pyroptosis is an attractive cell death mechanism for inducing anti-tumor responses in specific cancer types [4]. Chemotherapeutic drugs can induce GSDME-mediated pyroptosis and thus may contribute to beneficial responses in a subset of cancer patients [8]. In this study, we investigated the ability of GB cells to undergo GSDME-mediated pyroptotic cell death. First, we analyzed *GSDME* expression in public databases, using spatial transcriptomics of patient biopsies as well as GB cell lines. In contrast to most other cancer types, where *GSDME* is either mutated or expressed at low levels [4], we found high expression levels of GSDME in patient samples and GB cell lines. A previous study also reported high *GSDME* expression in GB [17]; however, it did not include an analysis of GSC lines. To investigate if the pyroptotic machinery is intact in GB, we treated human and mouse lines with the drug raptinal, which releases cytochrome c into the cytoplasm and activates the intrinsic apoptotic cascade [13, 15]. Vernon et al. demonstrated that raptinal induced GSDME cleavage downstream of caspase 3, promoting pyroptosis in melanoma [14]. Here, we confirm cytochrome c release, caspase 3, and GSDME cleavage in human as well as mouse GB cells. When analyzing pyroptosis, we surprisingly found differences in the pyroptotic efficacy between human and mouse GB lines. While mouse lines were susceptible to pyroptotic cell death, as confirmed by morphology and PI uptake, human cells showed resistance towards pyroptosis. This resistance was partially reversed by using the calcium-chelator BAPTA-AM, which may indicate active membrane repair mechanisms. Previously, it has been shown that active plasma membrane repair is a critical resistance mechanism for pyroptosis induction [16]. However, as calcium is involved in many cellular processes, we cannot exclude that other calcium-dependent mechanisms influencing cell death were affected. Our results stay in contrast to a study by Fang et al. demonstrating efficient pyroptosis in GB models [17]. However, models used in their study are based on serum-cultured cell lines, which do not very closely reflect the geno- and pheno-type of GB patient samples [18]. In our study, we used GSC lines, which are widely accepted as representative models of GB in patients [18–21]. As mouse GB lines were susceptible to GSDME-mediated pyroptosis in vitro, we applied raptinal treatment in vivo in the CT2A model, which in vitro showed the most efficient pyroptosis induction. Raptinal is a small molecule, and according to ADMET predictions, it should pass the blood-brain barrier [22]. Raptinal is not toxic in vivo and showed therapeutic efficacy in a melanoma model by activating GSDME-mediated pyroptosis [14]. Surprisingly, in our study, raptinal treatment in the mouse GB CT2A model did not show a significant impact on survival. This stays in contrast to a study by Fang et al. delivering nanoparticles with the natural compound Aloe-emodin to GL261 glioma in mice [17]. The authors demonstrated GSDME cleavage, a significant treatment effect, and activation of anti-tumor immunity. However, they did not verify whether this effect was dependent on GSDME-mediated pyroptosis by using, for example, knockout experiments. In our study, we performed knockout of the *Gsdme* gene in mouse GB cells and demonstrated that pyroptosis can be inhibited upon raptinal treatment in vitro. However, *Gsdme* knockout in vivo prolonged survival of mice, which was independent of raptinal treatment and thus the pyroptotic function of GSDME. Analysis of the immune microenvironment revealed a significant increase in CD4+, CD8+, and Granzyme B + T cells. These results indicated a tumor-promoting function of GSDME. We confirmed these results by spatial analysis of GB patient samples and gene expression correlation in TCGA data. Interestingly, a similar negative impact of GSDME on immune cell infiltration was observed in a study on head and neck squamous cell carcinoma, where the authors found that *GSDME* expression levels negatively correlated with CD8+ T cell and B cell infiltration [23]. In contrast, Zhang et al. showed that GSDME-mediated pyroptosis in melanoma cells has a tumor-suppressing function by enhancing the number and functions of NK cells and CD8+ cytotoxic T cells. GSDME-mediated pyroptosis is further enhanced through Granzyme B released by activated NK cells [4]. This discrepancy of results between different studies highlights that the function of GSDME may be highly dependent on the cancer type. This also correlates to the endogenous expression/mutations found in different cancers. GSDME most likely has a tumor-suppressing function in cancers where GSDME is frequently mutated, while it has the opposite effect in cancer types with high endogenous expression of GSDME, such as GB.

As *Gsdme* KO in GB increased the survival rate of mice in our study, we speculated that GSDME might affect cell proliferation and invasion of glioma cells. Cell cycle analysis revealed no difference between *Gsdme*/*GSDME* knockout and ctrl cells. However, wound healing assays and collagen invasion assays showed a decrease of invasion upon *Gsdme*/GSDME knockout in both mice and human GB.

Future studies are needed to provide mechanistic insights into the pro-tumorigenic functions of GSDME in GB. Our study is not the first to describe a tumor-promoting role of GSDME independent of its pyroptotic function in cancer. Lv et al. demonstrated that GSDME mediated protection of pancreatic adenocarcinoma from enzymatic digestion involving the YBX1-mucin pathway [24].

In summary, our study shows that GB’s molecular machinery for GSDME-mediated pyroptosis is intact. However, there is profound resistance to pyroptosis in human GB cells, which may be mediated by active plasma membrane repair mechanisms. Further research is needed to understand resistance mechanisms. We demonstrate that GSDME has tumor-promoting functions independent of pyroptosis, including suppression of T cell infiltration and invasion of GB cells. Our study indicates that pyroptosis induction by GSDME may be a difficult future avenue for clinical translation in GB. Instead, targeting GSDME as a tumor promoter may be a more promising strategy.

## Methods

### TCGA dataset analysis

The TCGA_GB samples were selected in the GlioVis portal [25]. Scaled Log2 expression of *Deafness autosomal dominant 5*, *DFNA5* (*GSDME*), was compared between tumor and non-tumor samples within the dataset. In the IVY_GAP dataset curated within the GlioVis portal, Log2 expression *GSDME* was compared between the different histological regions within the dataset. Pearson correlation analysis was also performed between *GSDME* and *CD3D, CD8A, CD8B*, and *Granzyme B*. *GSDME* analysis across cancer types was derived from proteinatlas.org [10] and was performed using GlioVis, with the adult TCGA_GBM dataset on the HG-U133A platform.

### scRNA-seq data analysis

For the scRNA-seq data analysis, we used the curated publicly available GBmap dataset with data from 240 *IDH* wild-type GB patients [26]. Individual cells were scored for the expression of the different GB cellular states using the marker list for each cellular state and plotted in quadrants as described by Neftel et al. [27]. *GSDME* expression was plotted as color gradient.

We reanalyzed the scRNA-seq datasets published in Darmanis et al. [11] from *IDH* wild-type GB (*n* = 4) to identify the expression of *GSDME* in different cell types within the GB TME. The sample processing techniques of the dataset allow us to see the expression in different tumor regions (Tumor core, periphery, and distant regions). Tumor region and cell type information were obtained from the dataset’s metadata. *GSDME* gene expression data were extracted from the Darmanis dataset using the FetchData() function. The extracted *GSDME* expression values, cell type and tumor region information were combined into a single dataframe, which was then used for visualization.

Summary Statistics: The mean expression and standard error (SE) of *GSDME* for each cell type were calculated. A bar plot was generated to show the mean *GSDME* expression for each cell type, with error bars representing the standard error.

### Spatial transcriptomics data

Spatial transcriptomics data were generated as described in Ravi et al. [2]. Briefly, Spatial transcriptomics experiments were conducted on *IDH* wild-type GB tumors using the 10X Visium Spatial Gene Expression kit, following the manufacturer’s protocol for tissue optimization and library preparation. Fresh tissue samples were collected immediately after resection, embedded in Tissue-Tek O.C.T. Compound, and snap-frozen in isopentane cooled with liquid nitrogen. The tissue was stored at −80 °C until further processing. Ten-micrometer tissue sections per sample were lysed using TriZol, and RNA was isolated using the PicoPure RNA Isolation Kit. RNA integrity was assessed using a Fragment Analyzer, and only samples with an RNA integrity number (RIN) greater than 7 were used for further analysis.

For the spatial gene expression protocol, 10 µm tissue sections were mounted on spatially barcoded glass slides with poly-T reverse transcription primers, one per array, and fixed with 100% methanol. H&E staining and brightfield imaging were performed at 10X magnification, followed by permeabilization to capture mRNA on the primers. cDNA was generated using template switch oligos and amplified with KAPA SYBR FAST qPCR Master Mix. After size selection using SPRIselect reagent, quality control was performed with a Fragment Analyzer. The cDNA was further optimized for sequencing using Illumina NextSeq, with unique indexes and Illumina primers added. Final libraries were quantified and sequenced on the Illumina NextSeq 550 platform, using paired-end sequencing with 28 cycles for reading 1, 10 cycles per index, and 120 cycles for read 2 on a NextSeq 500/550 High Output Kit v2.5 (Illumina, 20024907).

### Spatial transcriptomics data analysis

The data was processed using the SPATA2 pipeline [28]. SPATA2 objects were scaled-normalized and denoised using autoencoder denoising, and spots were annotated using Cell2location algorithm. Spatial transcriptomics of *IDH* wild-type GB patients (*n* = 10) obtained from the Visium platform were processed using SPATA2. The clinical data of the patients are presented in Supplementary Table 3. A list of 10 SPATA objects was created, each representing spatial transcriptomic data for different GB samples. To investigate the spatial relationships between cells expressing *GSDME* and T cells, we used the expression of key marker genes to define Tumor cells (*EGFR, SOX2, GFAP*, and *MKI67*), *CD4* (*CD3D*), and *CD8* (*CD8A*) T cells. Moran’s I was computed for spatial autocorrelation using getMoransI function from the SPATIAWrappers package (Supplementary Table 4). The average Moran’s I was calculated for each feature and represented in matrix form. Spatial correlation was calculated using MERINGUE to quantify the relationship between gene expression and spatial proximity of cells. The average spatial correlation matrix was calculated by summing the individual spatial correlation matrices and averaging the values. This matrix was visualized using ggcorrplot.

### Cell culture

P3, BG5, and BG7 are patient-derived GB stem cell (GSC) lines that were established from patients with *IDH* wild-type GB [29]. ‘Complete’ Neurobasal medium (NBM) was used for the culture of GSC lines: Neurobasal medium (Gibco, MA, USA), 2% B-27 supplement, minus vitamin A (Gibco), 1% L-glutamine (Sigma-Aldrich, MO, USA), 1% Penicillin-Streptomycin (Sigma-Aldrich) 20 ng ml^−1^ epidermal growth factor (EGF) (PeproTech, NJ, USA), 20 ng ml^–1^ fibroblast growth factor 2 (FGF-2) (PeproTech) and 0.2% heparin (LEO Pharma, NOR). P3 cells were cultured in media without EGF.

CT2A and GL261 are frequently used mouse glioma cell lines [30]. The CT2A cell line was kindly received from Prof. Kathrin Lamszus, University Clinic Eppendorf, Hamburg, Germany. The GL261 cell line was obtained from the National Cancer Institute-Frederick Cancer Research Tumor Repository. These cell lines were cultured in Dulbecco’s Modified Eagle’s medium (DMEM; Sigma-Aldrich) supplemented with 10% Fetal Bovine Serum (FBS; Sigma-Aldrich), 2% L-glutamine (Sigma-Aldrich), 2% Penicillin-Streptomycin (Sigma-Aldrich), 5 mg/ml plasmocin (InvivoGen, CA, USA), and 3.2% non-essential amino acids (NEAA; Biowest, France).

All cell lines were maintained in an incubator regulated at 37 °C, 100% humidity, and 5% CO_2_.

For in vitro experiments, cells were treated with 10 μM raptinal (Sigma-Aldrich) at time points ranging from 15 min to 6 h. The raptinal stocks were prepared by dissolving raptinal in DMSO to a concentration of 25 mM.

### Generation of *Gsdme*/*GSDME* knockout cells

Mouse *Gsdme*/Human *GSDME* gRNA sequences have been published previously [4]. gRNA oligos were synthesized and cloned into lentiCRISPRv2 puro (Brett Stringer Lab, addgene, AUS) as previously described [31, 32].

Successful cloning was confirmed by sequencing. Lentiviral particles encoding the *Gsdme*/*GSDME* gRNA sequences were prepared, and target cells were transduced according to a protocol as described previously [33]. Transduced cells underwent puromycin (Thermo Fisher, MA, USA) selection (1.5 µg/mL for mouse cell lines and 1 µg/mL for human cell lines) until all control cells died. Puromycin media was refreshed every other day. The bulk knockout population was seeded at 0.5 cells per 200 μl in 96-well plates for single-cell clonal selection. Clonal populations were screened by immunoblotting for *Gsdme*/*GSDME* expression, and clones showing complete protein depletion were selected for subsequent experiments.

### Western blotting

The cells were washed once with phosphate-buffered saline (PBS). Cell pellets were lysed with ice-cold Mammalian Protein Extraction Reagent (M-PER; Thermo Scientific) and enriched with 1% protease inhibitor and 1% phosphatase inhibitor cocktails (Roche, CH). Pierce BCA Protein Assay Kits (Thermo Scientific) were used to determine the protein concentration of the lysates. The reagents for the NuPage System (Invitrogen, MA, USA) were utilized according to manufacturer instructions. Denatured protein samples were loaded on SDS page 12% Bis-tris gel and transferred to Immobilon-NC Transfer Membrane (Millipore, MA, USA). Membranes were blocked with 5% skim milk TBS-Tween solution to prevent unspecific binding. Blocking was followed by overnight incubation at 4 °C with the primary antibody and 1 h incubation at room temperature with the secondary antibody. The primary and secondary antibodies used are listed in Supplementary Table 1. The membranes were developed with SuperSignal™ West Pico PLUS Chemiluminescence Substrate (Thermo Scientific) by using LAS 3000 (version 2.2; Fujifilm, JP) and a ChemiDoc (Bio-Rad, CA, USA).

### Cytochrome C release assay

The cells were washed once with PBS. The pellets were first lysed with digitonin permeabilization buffer (190 μg/ml digitonin, 1 mM sodium phosphate monobasic, 8 mM sodium phosphate dibasic, 75 mM NaCl, 250 mM sucrose), enriched with protease and phosphatase inhibitor cocktails, and maintained at pH 7.5 to isolate the cytosolic fraction. The remaining pellets were washed with digitonin permeabilization buffer and lysed with M-PER (BG5 and BG7) or RIPA Lysis and Extraction Buffer (Thermo Scientific) (P3, GL261, and CT2A) enriched with protease and phosphatase inhibitor cocktails to isolate the mitochondrial fraction. Anti-cytochrome c (Invitrogen) was the primary antibody. Beta-actin (Abcam, UK) and COX IV (Abcam) were loading controls for the cytosolic and mitochondrial lysates. LAS 3000 was used to develop the membranes.

### WST-1 cell viability assay

Cells were seeded into flat-bottom 96-well plate(s) at a density of 1.0 × 10^4^/100 μl. P3 and CT2A cells were seeded at a density of 1.5 × 104/100 μl. After incubation overnight, the cells were treated with 10 μM raptinal for 0.5, 1, 2, and 3 h (P3, BG5, and BG7) and 0.5, 1, 2, 3, and 6 h (CT2A and Gl261). Following treatment, 7 μl WST-1 reagent (Roche) was added to the wells. After incubating for 90 min, the absorbance was measured with a Multiscan FC Microplate Photometer (Thermo Fisher). The relative viability was plotted in GraphPad Prism.

WST-1 assay was performed with human cells using 4 technical replicates (*n* = 4), and with mouse cells using 6 technical replicates (*n* = 6).

### In vivo experiments

C57BL/6 mice, procured from Janvier, were housed under standard conditions with ad libitum access to food and water. All procedures followed the Norwegian Animal Act and were approved by the local ethics committee. Syngeneic tumor cells were implanted orthotopically following a protocol described previously [34]. Tumor progression was monitored weekly using MRI, with imaging parameters as previously described [34]. After tumor establishment, raptinal or the vehicle (10% DMSO in trans-fat-free corn oil (Sigma-Aldrich)) was administered intraperitoneally (300 μL, 20 mg/kg) once daily for four consecutive days [13]

The study included the following experimental groups: Vehicle (*n* = 4), Raptinal (*n* = 7), Vehicle *Gsdme* KO (*n* = 7), and Raptinal *Gsdme* KO (*n* = 8).

### Immunohistochemistry (IHC) and image analysis

IHC of formalin-fixed paraffin-embedded (FFPE) brain sections was carried out as previously described [19]. Primary and secondary antibodies are listed in Supplementary Table 2. IHC stainings included the following experimental groups: Vehicle (*n* = 3), Raptinal (*n* = 6), Vehicle *Gsdme* KO (*n* = 6), and Raptinal *Gsdme* KO (*n* = 8).

Whole slide images were acquired using an Olympus V120 slide scanner (Olympus Corporation, Japan). Using QuPath version 0.51.1 [35], a neural network pixel classifier was trained to separate the image into tumor, stroma, and background/non-tissue. These preliminary classifications were then manually optimized. QuPath was then used to segment the tumor regions into cells. Color deconvolution, along with a fixed threshold for DAB intensity, was used to define positive cells.

### Cell cycle analysis

Cells were seeded into T25 flasks at a density of 2 × 10^6^/4 ml (CT2A) or 1 × 10^6^/4 ml (GL261 and P3). Following harvesting, the pellets were fixed by adding 70% ice-cold ethanol while vortexing, followed by double washing step with PBS. The pellets were resuspended in 500 μl of Propidium Iodide (BioLegend, CA, USA) staining solution (50 μg/mL PI and 100 μg/mL RNase A (Thermo Scientific) in PBS) and incubated in the dark at 37 °C for 30 min. The cell distributions were analyzed by flow cytometry for 25,000 events (Accuri C6, BD Biosciences, NJ, USA) and FlowJo v10.9. Cell cycle analysis was performed with three technical replicates (*n* = 3) for all cell lines.

### Collagen invasion assay

Two days before starting the assay, tumor cell spheroids were prepared by seeding 5000 cells in culture media containing 0.04% methylcellulose (Sigma-Aldrich) in a low attachment U-bottom 96-well plate (Costar, Corning, NY, USA). The collagen matrix containing 1 mg/ml of collagen I (Corning) was prepared using cold PBS with 7.2 mM NaOH. The complete collagen matrix was incubated on ice for 30 min. The spheroids were washed in PBS twice, placed in 100 μl collagen matrix, and transferred to a flat-bottom 96-well plate. After 30 min incubation at 37 °C, media was added to the wells. 24 h, 48 h, and 72 h images were captured by Nikon Ti2 Eclipse (Nikon, JP). Invasion distance was measured using NIS Element software. The distance from the center to the five furthest invasive cells for each spheroid was measured and averaged, with satellite cells excluded from the analysis. Each experiment consisted of 3–5 (*n* = 3–5) technical replicates per condition (varying due to spheroid integrity) for all cell lines.

### Wound healing assay

The wound healing assay was employed for the GL261 and GL261 *Gsdme* KO cell lines. A flat-bottom Incucyte Imagelock 96-well Microplate (Sartorius, DE) was used. The cells were seeded at a density of 5.0 × 10^5^/200 μl. The scratch wounds were made by MIC facility personnel at the University of Bergen according to a protocol provided by Sartorius. Wound healing assay was performed with using 16 technical replicates (*n* = 16) for each cell lines.

### PI uptake assay

For Incucyte imaging and propidium iodide (PI) uptake assay, cells were seeded into flat-bottom 24-well plates with a 5.0 × 10^5^/1 ml density. CT2A cells were seeded with a density of 7.5 × 10^5^/1 ml. P3, BG5, and BG7 cells were seeded on 2% Matrigel Matrix (Corning) coated plates. After overnight incubation, the media in each well was discarded and replaced by PI-containing media (2.5 μg/ml; BioLegend). The cells were then treated by adding raptinal prepared in PI media. For BAPTA-AM (MedChemExpress, NJ, USA) treatment, P3, BG5, and BG7 cells were treated with BAPTA-AM (5 µM, 2.5 µM, 2.5 µM) for 1 h before the assay start. Incucyte S3 Live-Cell Analysis System (Sartorius) was used for real-time monitoring and analysis of cells. PI uptake assay was performed with human cells using 4 technical replicates (*n* = 4), and with mouse cells using 2 technical replicates (*n* = 2).

### Incucyte imaging and quantification

Following overnight incubation post-seeding, the cells were treated with 10 μM raptinal for 6 h, and each well was imaged with Incucyte S3 (Sartorius) in four-set positions every 1 h.

Two distinct analyses were performed on the same experiments: PI uptake and pyroptotic morphology. The number of cells displaying pyroptotic morphology was manually quantified using NIH ImageJ software, while PI uptake was measured using Incucyte software. Pyroptotic morphology was identified by the presence of bubble-like protrusions on the cell surface [36]. PI uptake, indicating pore formation in the cell membrane, was visualized as a significant color change in the affected cells. For each image quantified, the percentage of pyroptotic cells was calculated. Total PI uptake was measured by total red object integrated intensity (RCU × µm^2^/image).

### Statistics and reproducibility

Most of the in vitro experiments described in this study utilized a minimum of three independent biological replicates. Survival analysis was conducted using the Kaplan–Meier estimator with a Log-Rank test in GraphPad Prism software. Significant 2-way ANOVA or Ordinary one-way ANOVA results were followed by post-hoc pairwise comparisons, applying Tukey’s or Dunnett’s multiple comparison test. The alpha threshold for statistical significance was set at (*p* < 0.05). The *p* values indicating statistical significance are: (*p < 0.05); (**p < 0.01); (***p < 0.001); (***p < 0.0001). Values are presented as means ± standard error of the mean (SEM).

Statistical analysis of IHC image analysis data was performed using RStudio software (RStudio 2023.06.1 + 524) and generalized linear models with the glm function. Primary analysis comparing *Gsdme* KO to control in the animal experiment was done using a Wilcoxon rank-sum test. For the sensitivity analysis, generalized linear models were fit with predictors raptinal, KO, and their interaction. Based on whether the dependent variable took the form of counts or percentages, a quasipoisson distribution and log link function or quasibinomial distribution and logit link function was used.

## Supplementary information

Supplementary Figure legends

Supplementary Figure 1

Supplementary Figure 2

Supplementary Figure 3

Supplementary Figure 4

Supplementary Figure 5

Supplementary original Western blots

Supplementary Tables 1-4

## Data Availability

The original contributions presented in this study are included in the article/supplementary material. Further inquiries can be directed at the corresponding author(s).
