# Supplementary material for: Gasdermin E in glioblastoma –pyroptosis resistance and tumor-promoting functions
Source: Cell Death Discov. 2025 Jun 21;11:284. doi: 10.1038/s41420-025-02572-z (PMC12182582; doi:10.1038/s41420-025-02572-z)
Supplement: Supplementary file 1 — Supplementary Figure legends [file 41420_2025_2572_MOESM1_ESM.docx]

**Supplementary Figure legends**

**Supplementary Figure 1: *GSDME* expression in GB is not influenced by hypoxia and is not different between cellular states. A)** *GSDME* expression is higher in the tumor core as compared to tumor periphery or distant areas. Data derived Darmanis et al. (1) **B)** Expression analysis of *GSDME* in GB subtypes according to Neftel et al. (2), derived from scRNA seq data from the GBMap dataset (3). **C)** Western blot of GSDME (full length and cleaved (clvd)) in human GSC lines and mouse GB lines under normoxic and hypoxic (1% O_2_) conditions. Treatment of A549 with 10 µm raptinal, an inducer of apoptosis/pyroptosis was used as a control for clvd GSDME. β-Actin was used as a loading control. **D)** Surface and gradient plots showing the distance of *GSDME* expression around a hypoxic region in a GB patient.

**Supplementary Figure 2: Sensitivity of cancer cells to raptinal and cytochrome c release upon raptinal treatment in GB cells. A)** WST-1 assay of different cancer cell lines treated with 10 μM raptinal. Time points after the start of treatment are indicated. Data represented as mean ± SEM (n=5-6). * (and corresponding signs) p<0.05; ** (and corresponding signs) p<0.01; *** (and corresponding signs) p<0.001; *** (and corresponding signs) p<0.0001. **B)** Western blot of cytosolic and mitochondrial cytochrome C in BG5 cells treated with 10 μM raptinal. COXIV was used as a mitochondrial loading control and β-actin as a cytosolic loading control. **C)** Western blot for cleaved (clvd) Caspase 3, GSDME, and clvd GSDME in A549 and B16F10 cell lines treated with 10 μM raptinal. Time points after the start of treatment are indicated. β-actin was used as loading control.

**Supplementary Figure 3: Mouse GB CT2A are very sensitive to pyroptosis induction by raptinal treatment. A)** Pyroptosis induction in BG7 cells was quantified at the indicated time points post-raptinal treatment by microscopy, combining morphology and PI uptake. Data represented as mean ± SEM (N=3). ** p<0.01; **** p<0.0001. The scale bar indicates 200 µm. **B)** Pyroptotic morphology comparison of human GB cell lines and mouse GB cell lines at the indicated time points post-raptinal treatment. Data represented as mean ± SEM (N=3). * p<0.05; ** p<0.01; *** p<0.001; **** p<0.0001 **C)** Western blot for GSDME in CT2A, GL261 and P3 ctrl and KO clones. Clones used in this study are marked by *. **D)** Pyroptosis was measured by PI uptake in mouse GB ctrl and *Gsdme* KO cell lines post-raptinal treatment at the indicated time points. Data represented as mean ± SEM (N=3). * p<0.05; ** p<0.01; *** p<0.001; **** p<0.0001. **E)** WST-1 assay of ctrl and *Gsdme* KO mouse GB lines treated with 10 μM raptinal. Time points after the start of treatment are indicated. Data represented as mean ± SEM (N=3). * p<0.05; *** p<0.001. **F)** Pyroptosis was measured by PI uptake in BG7 cells post-raptinal treatment at the indicated time points. BAPTA-AM was used to block plasma membrane repair mechanisms. Data represented as mean ± SEM (N=3). * p<0.05; ** p<0.01; **** p<0.0001.

**Supplementary Figure 4: GSDME inhibits T cell infiltration in GB. A)** Quantifications of IHC stainings from the mouse experiment (Figure 4A-C) are displayed for each group. Multivariate sensitivity analyses were performed and are shown in the bar graph. All data represented as mean ± SEM, (n=3-8). In addition, a summary figure and table are shown. The (Intercept) estimates the average number of positive cells in the vehicle/ctrl group, while the other numbers describe the difference from this baseline. * p<0.05; ** p<0.01; *** p<0.001. **B**) H&E staining and spatial annotation of tissue section from patient (269UKF) used for spatial transcriptomics showing different histologic regions (tumor, transition, and infiltrated) in the tumor section. **C)** The correlation coefficient of *DFNA5(GSDME)* and CD3D, CD8A, CD8B, and Granzyme B from TCGA data is shown. Analysis was performed using GlioVis, with the adult TCGA_GBM dataset on the HG-U133A platform.

**Supplementary Figure 5: GSDME expression does not influence the cell cycle of GB cells but promotes migration of GL261 mouse GB cells. A)** Cell Cycle analysis of P3, GL261, and CT2A ctrl and *Gsdme/GSDME* KO cells by flow cytometry. Data represented as mean ± SEM (N=2) **B)** Wound healing assay with GL261 and GL261 *Gsdme* KO mouse GB cells measured at indicated time points. The scale bar indicates 400 µm.

**References**

1. Darmanis S, Sloan SA, Croote D, Mignardi M, Chernikova S, Samghababi P, et al. Single-Cell RNA-Seq Analysis of Infiltrating Neoplastic Cells at the Migrating Front of Human Glioblastoma. Cell Rep. 2017;21(5):1399-410.

2. Neftel C, Laffy J, Filbin MG, Hara T, Shore ME, Rahme GJ, et al. An Integrative Model of Cellular States, Plasticity, and Genetics for Glioblastoma. Cell. 2019;178(4):835-49 e21.

3. Ruiz-Moreno C, Salas SM, Samuelsson E, Brandner S, Kranendonk M, Nilsson M, et al. Harmonized Single-Cell Landscape, Intercellular Crosstalk and Tumor Architecture of Glioblastoma. Neuro-Oncology. 2022;24:287-.
