# Supplementary figures and images for: Gasdermin E in glioblastoma –pyroptosis resistance and tumor-promoting functions
Source: Cell Death Discov. 2025 Jun 21;11:284. doi: 10.1038/s41420-025-02572-z (PMC12182582; doi:10.1038/s41420-025-02572-z)

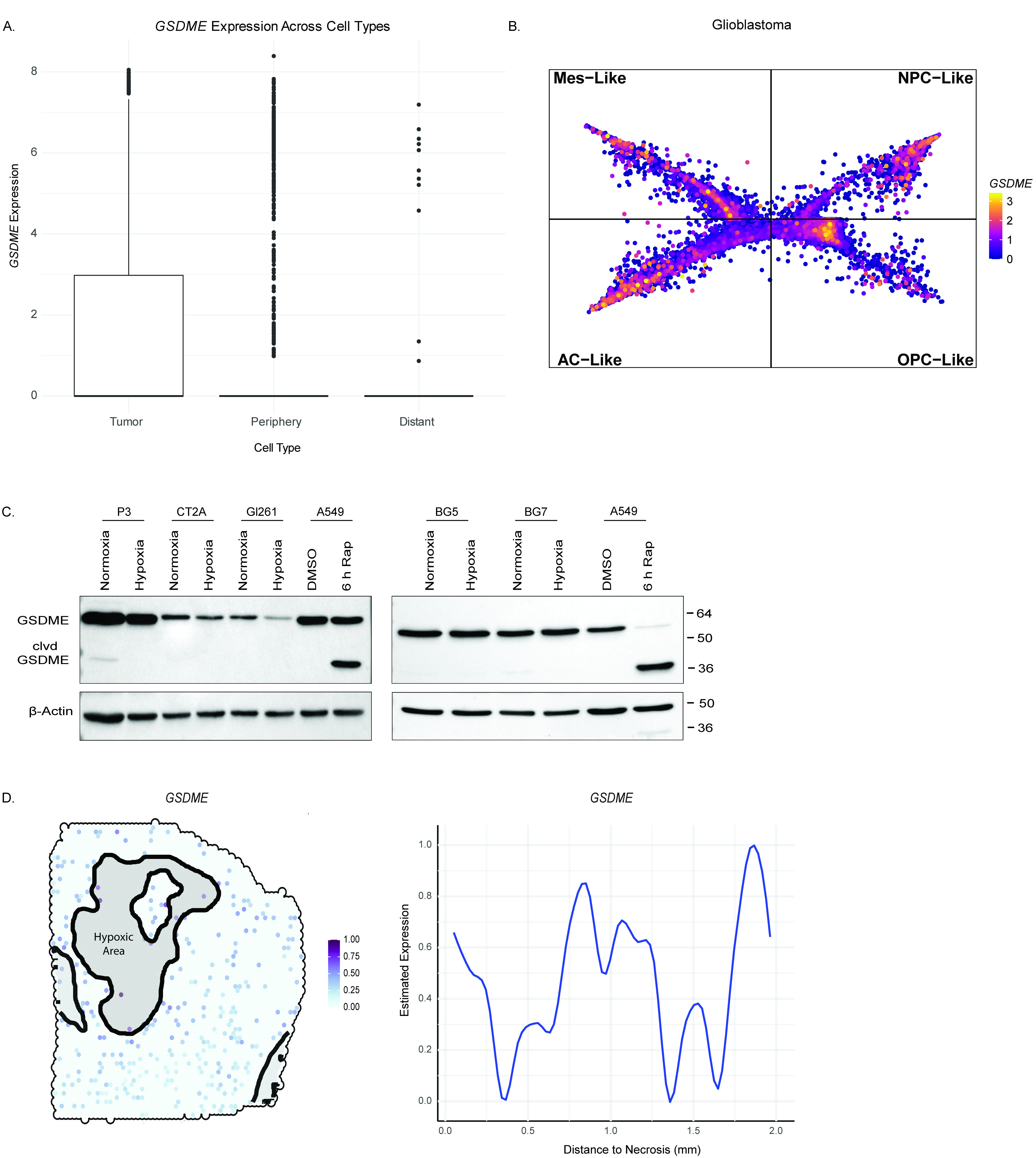

Supplement: Supplementary file 2 — Supplementary Figure 1 [file 41420_2025_2572_MOESM2_ESM.tif]

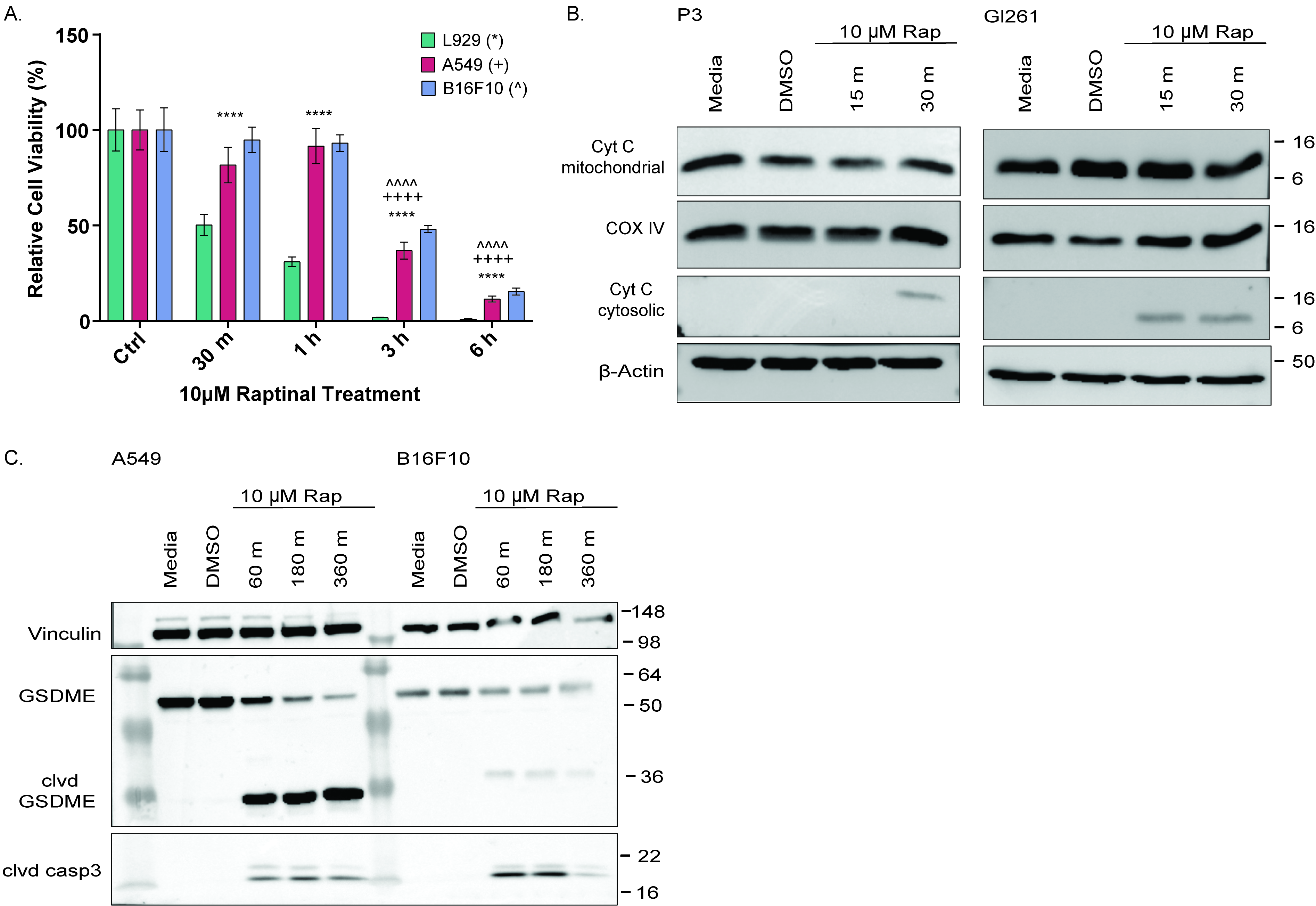

Supplement: Supplementary file 3 — Supplementary Figure 2 [file 41420_2025_2572_MOESM3_ESM.tif]

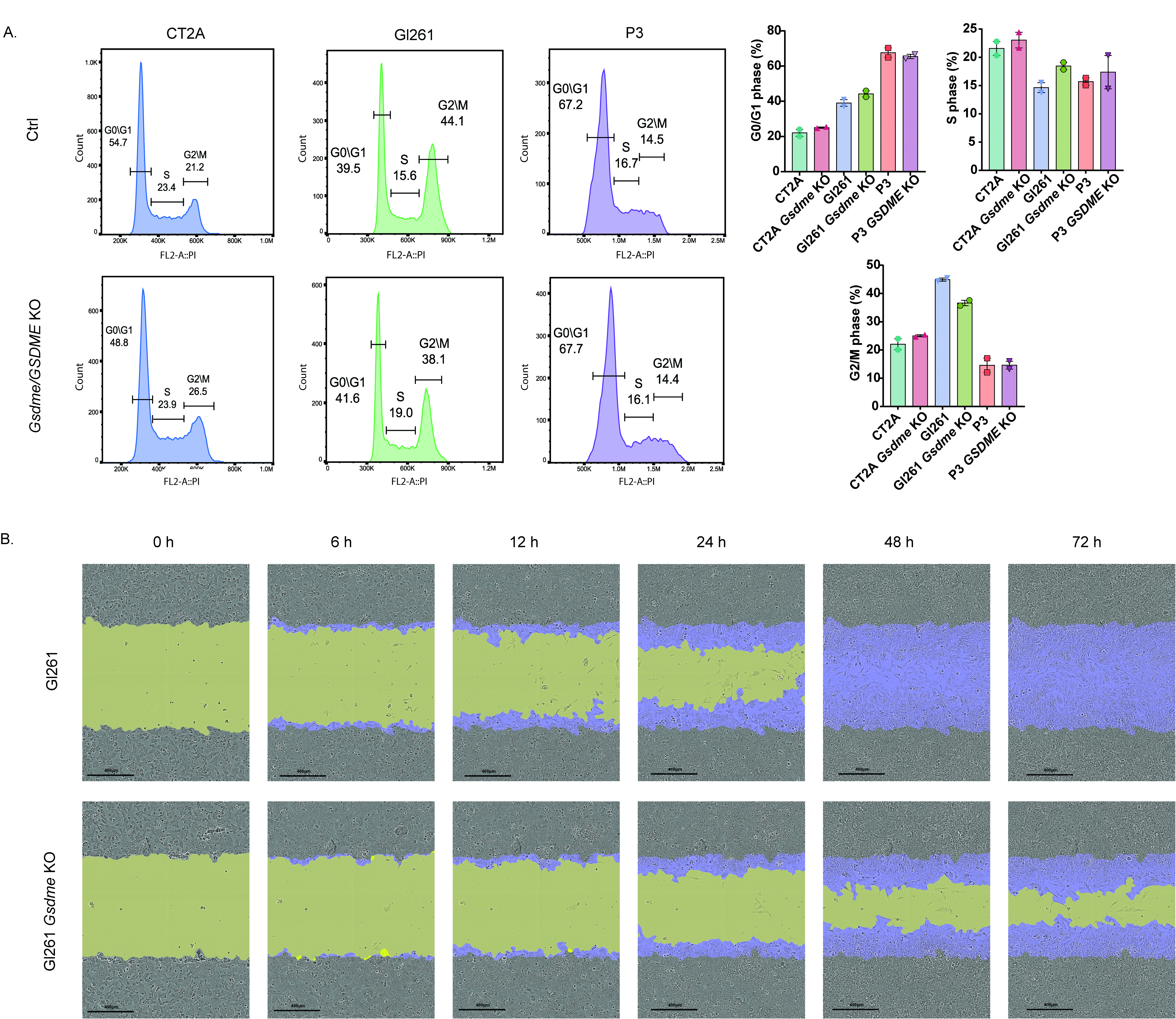

Supplement: Supplementary file 6 — Supplementary Figure 5 [file 41420_2025_2572_MOESM6_ESM.tif]

Figure 1D

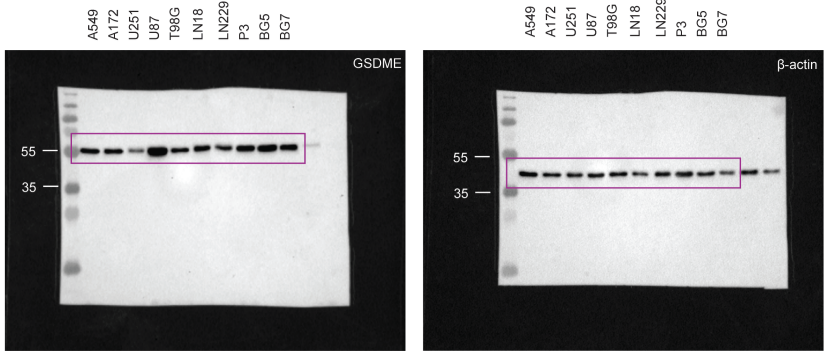

Figure 2C

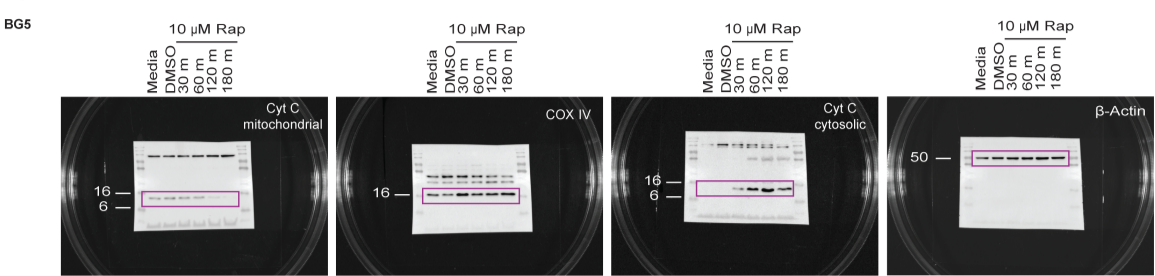

Figure 2D

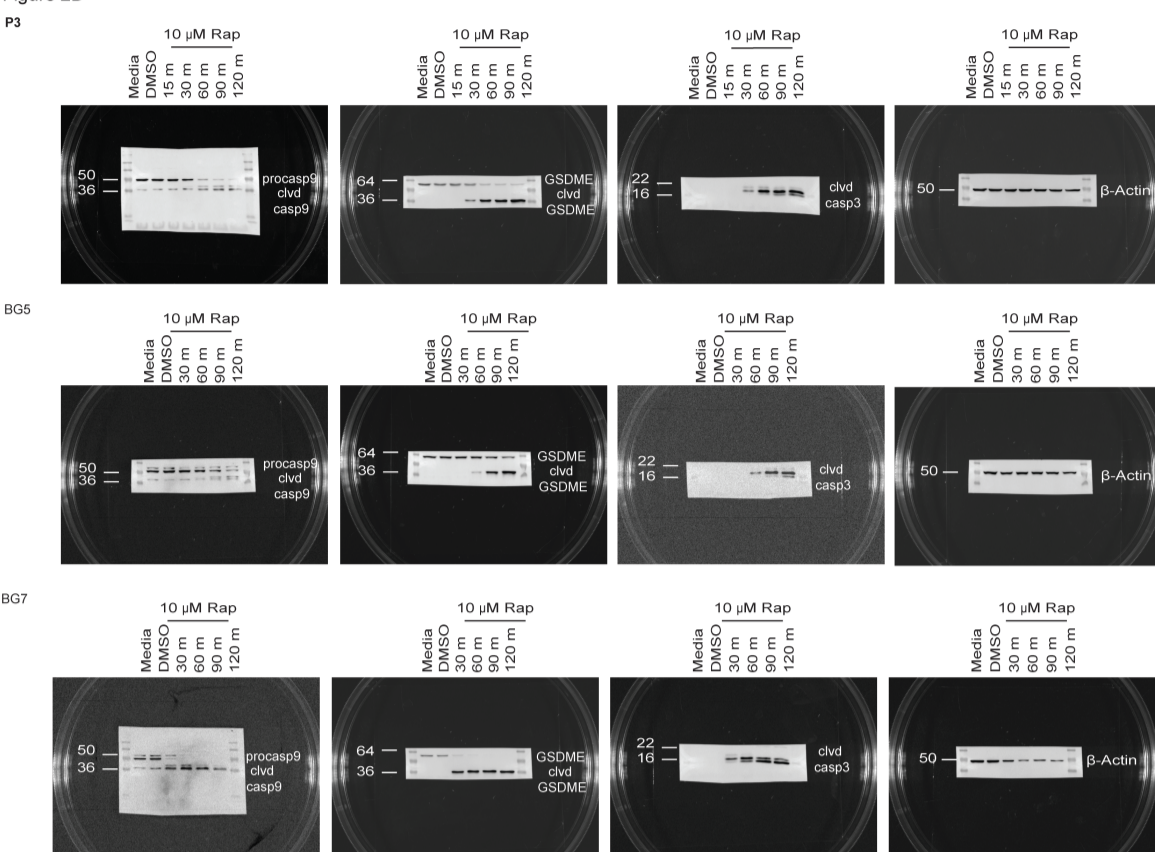

Figure 2E

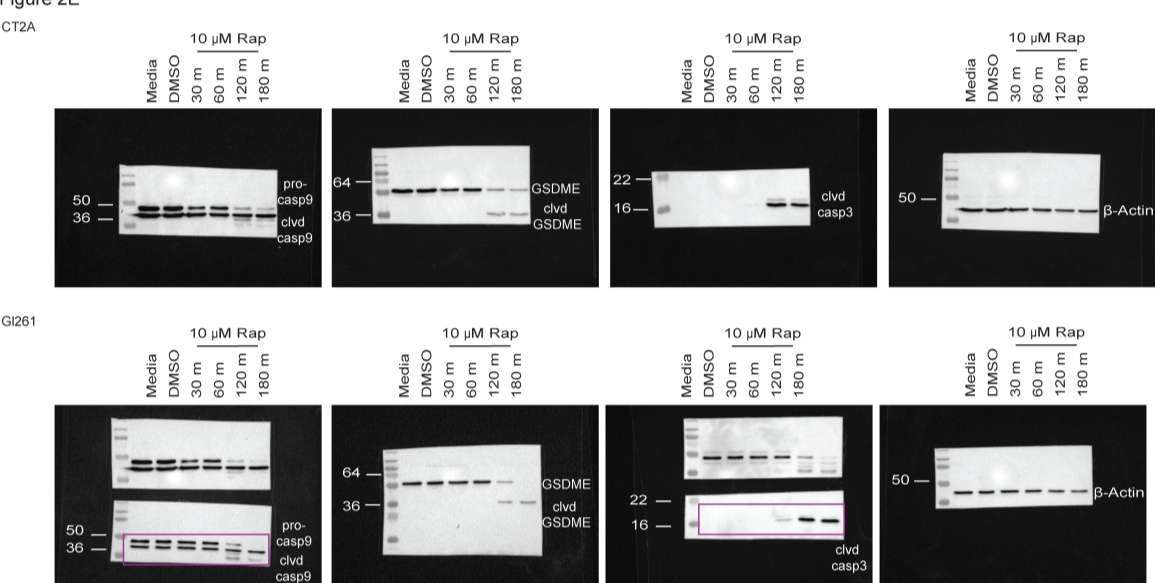

Suppl. Figure 1C

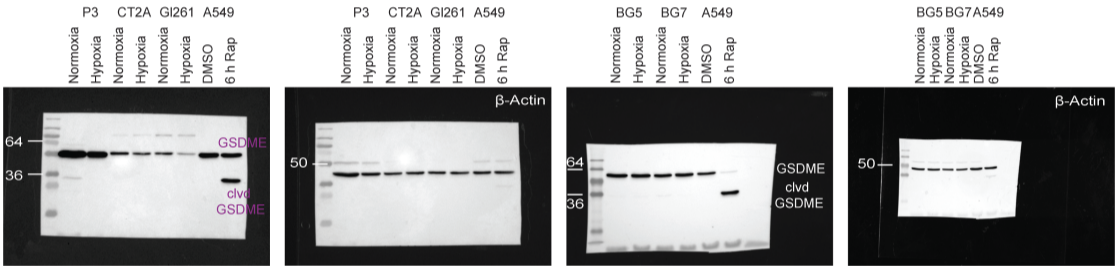

Suppl. Figure 2B

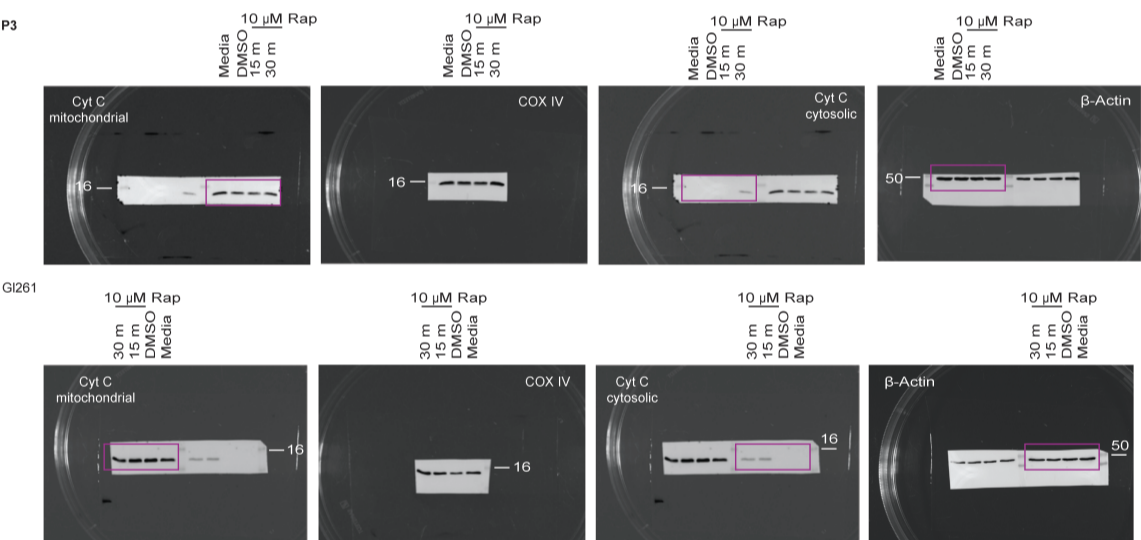

Suppl. Figure 2C

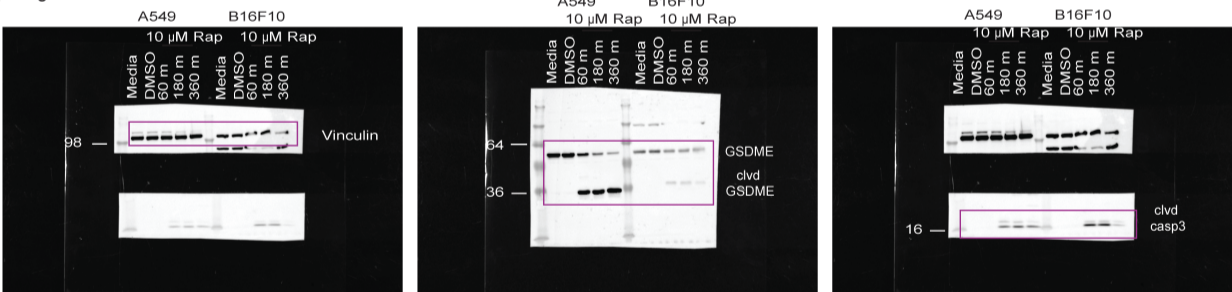

Suppl. Figure 3C

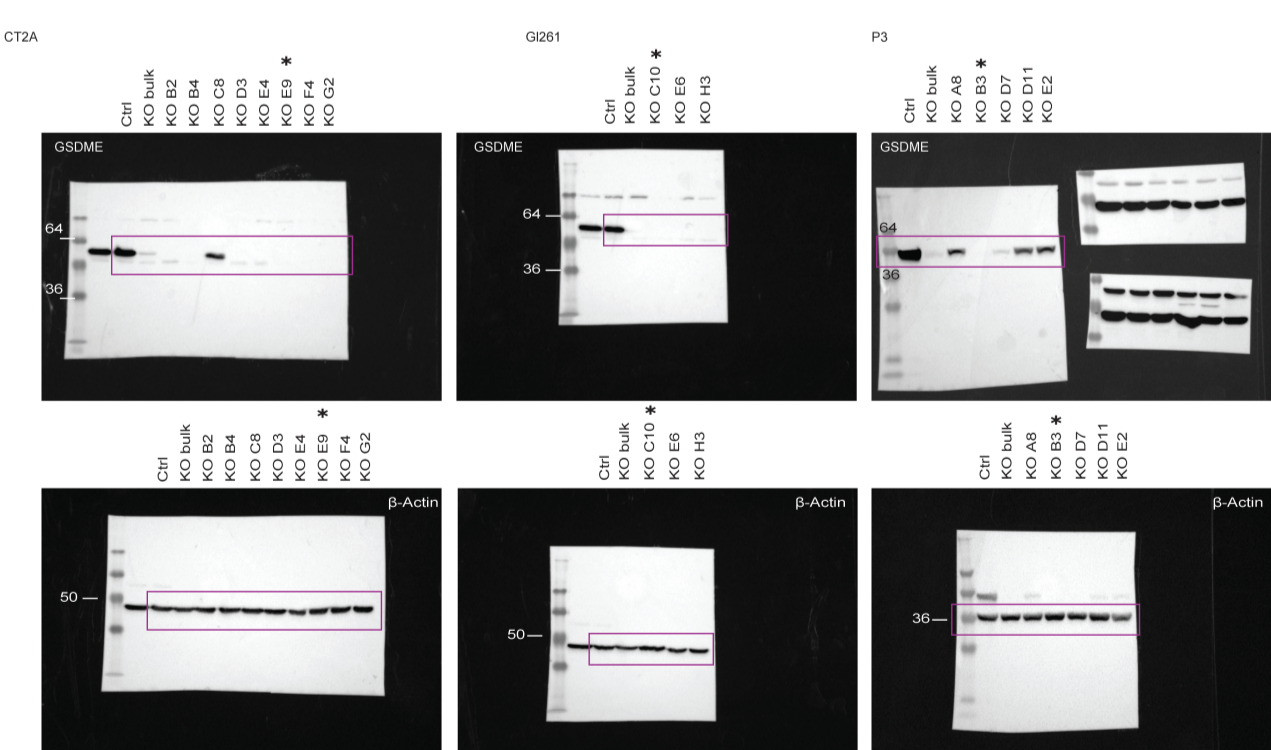

Supplement: Supplementary file 7 — Supplementary original Western blots [file 41420_2025_2572_MOESM7_ESM.pdf]
