# Supplementary material for: Gasdermin E in glioblastoma –pyroptosis resistance and tumor-promoting functions
Source: Cell Death Discov. 2025 Jun 21;11:284. doi: 10.1038/s41420-025-02572-z (PMC12182582; doi:10.1038/s41420-025-02572-z)
Supplement: Supplementary file 8 — Supplementary Tables 1-4 [file 41420_2025_2572_MOESM8_ESM.docx]

Supplementary Table 1

Western blot antibodies

| Antibody | Dilution | Catalog # | Company |
| --- | --- | --- | --- |
| Cytochome C | 1:500 | MA5-11674 | Invitrogen |
| Caspase 9 | 1:1000 | 9508T | Cell Signaling |
| Caspase 3 | 1:1000 | 9664S | Cell Signaling |
| Caspase 8 | 1:1000 | MA141280 | Invitrogen |
| Gasdermin E | 1:1000 | Ab215191 | Abcam |
| Beta actin | 1:2000 | Ab8224 | Abcam |
| COX-IV | 1:2000 | Ab16056 | Abcam |
| Goat anti-Mouse IgG (H+L), HRP | 1:5000 | 31430 | Invitrogen |
| Goat anti-Rabbit IgG (H+L), HRP | 1:10000 | 31462 | Invitrogen |

Supplementary Table 2

IHC Antibodies

| Antibody | Dilution | Catalog # | Company |
| --- | --- | --- | --- |
| CD3 | 1:150 | ab11089 | Abcam |
| CD4 | 1:100 | ab183685 | Abcam |
| CD8 | 1:100 | ab217344 | Abcam |
| Granyzme B | 1:150 | ab4059 | Abcam |
| Ki67 | 1:100 | 14-5698-82 | Invitrogen |
| CD45 | 1:100 | sc53665 | Santa Cruz |
| F4/80 | 1:100 | sc52664 | Santa Cruz |
| Rabbit anti-Rat, mouse adsorbed (H+L), Biotinylated | 1:100 | BA-4001 | Vector Laboratories |
| Goat anti-Rabbit IgG (H+L), Biotinylated | 1:100 | BA-1000 | Vector Laboratories |

Supplementary Table 3

Clinical information of the cohort used for spatial transcriptomics

Patient ID Tissue type Brain region Age *IDH* status Diagnoses Sex

269UKF Tumor Occipital 51 *IDH*-WT GB M

313UKF Tumor Temporal 57 *IDH*-WT GB M

242UKF Tumor Frontal 81 *IDH*-WT GB F

243UKF Tumor Frontal 55 *IDH*-WT GB F

255UKF Tumor Temporal 81 *IDH*-WT GB F

266UKF Tumor Temporal 76 *IDH*-WT GB F

275UKF Tumor Frontal 80 *IDH*-WT GB F

296UKF Tumor Temporal 34 *IDH*-WT GB M

265UKF Tumor Temporal 55 *IDH*-WT GB M

262UKF Tumor Temporal 59 *IDH*-WT GB M

Supplementary Table 4

|  | Moran I | p_value |
| --- | --- | --- |
| ***GSDME*** | 0.36215497 | 0.00097561 |
| ***CD3D*** | 0.19306136 | 0.00097561 |
| ***CD3E*** | 0.04718747 | 0.00097561 |
| ***CD8A*** | 0.02963932 | 0.00097561 |
| ***GFAP*** | 0.10631853 | 0.00097561 |
| ***SOX2*** | 0.37546336 | 0.00097561 |
| ***MKI67*** | 0.29712676 | 0.00097561 |
| ***EGFR*** | 0.37554596 | 0.00097561 |
